# Rates of evolutionary change of resident *Escherichia coli* O157:H7 differ within the same ecological niche

**DOI:** 10.1186/s12864-022-08497-6

**Published:** 2022-04-07

**Authors:** Margaret D. Weinroth, Michael L. Clawson, Terrance M. Arthur, James E. Wells, Dayna M. Brichta-Harhay, Norval Strachan, James L. Bono

**Affiliations:** 1 U.S. Department of Agriculture, Agricultural Research Service, U.S. Meat Animal Research Center, Clay Center, NE 68933 USA; 2 Present address: U.S. Department of Agriculture, Agricultural Research Service, U.S. National Poultry Research Center, Athens, GA 30605 USA; 3 School of Biological Sciences, University of Aberdeen, Cruickshank Building, St Machar Drive, Aberdeen, Scotland AB24 3UU UK

**Keywords:** *Escherichia coli*, STEC, Feedlot, Cattle, WGS, Whole genome sequencing, Comparative genomics, Evolution, Foodborne outbreak

## Abstract

**Background:**

Shiga toxin-producing *Escherichia coli* (STEC) O157:H7 is a pathogen known to reside in cattle feedlots. This retrospective study examined 181 STEC O157:H7 strains collected over 23 years from a closed-system feedlot. All strains were subjected to short-read sequencing, with a subset of 36 also subjected to long-read sequencing.

**Results:**

Over 96% of the strains fell into four phylogenetically distinct clades. Clade membership was associated with multiple factors including *stx* composition and the alleles of a well-characterized polymorphism (*tir* 255 T > A). Small plasmids (2.7 to 40 kb) were found to be primarily clade specific. Within each clade, chromosomal rearrangements were observed along with a core phageome and clade specific phages. Across both core and mobile elements of the genome, multiple SNP alleles were in complete linkage disequilibrium across all strains within specific clades. Clade evolutionary rates varied between 0.9 and 2.8 SNP/genome/year with two *tir* A allele clades having the lowest evolutionary rates. Investigation into possible causes of the differing rates was not conclusive but revealed a synonymous based mutation in the DNA polymerase III of the fastest evolving clade. Phylogenetic trees generated through our bioinformatic pipeline versus the NCBI’s pathogen detection project were similar, with the two *tir* A allele clades matching individual NCBI SNP clusters, and the two *tir* T allele clades assigned to multiple closely-related SNP clusters.

**Conclusions:**

In one ecological niche, a diverse STEC O157:H7 population exhibited different rates of evolution that associated with SNP alleles in linkage disequilibrium in the core genome and mobile elements, including *tir* 255 T > A.

**Supplementary Information:**

The online version contains supplementary material available at 10.1186/s12864-022-08497-6.

## Background

Shiga toxin-producing *Escherichia coli* (STEC) O157:H7 are human foodborne pathogens of major public health concern [1, 2]. Symptoms of an infection range from mild self-limiting gastrointestinal symptoms to hemolytic-uremic syndrome (HUS), which can lead to acute renal failure and death [3]. Cattle are a major reservoir of STEC O157:H7, which are commensal in their microflora [4, 5]. Other sources of STEC O157:H7 include animals such as small ruminants, wildlife, and pigs, as well as leafy greens [5, 6]. While increases in processing control during beef harvest has helped reduce the burden of STEC O157:H7 [7], bovine associated outbreaks still occur [8, 9]. Work specific to understanding the ability of different STEC O157:H7 isolates to cause human disease has found an overrepresentation of the *tir* 255 T > A T allele (*tir* T) in human-derived isolates as opposed to the *tir* 255 T > A A allele (*tir* A), which were more highly represented in bovine derived samples, suggesting that *tir* T isolates have a higher propensity to cause disease [10].

Due to its ubiquity and ease of culture, *E. coli* has been subjected to a great deal of DNA sequencing; first, as one of the earliest bacteria to be subjected to whole genome sequencing in 1997 [11], followed by a pathogenic strain of STEC O157:H7 in 2001 [12]. Recently, characterization of *E. coli* has moved from single strain to comparative genomics and evolutionary studies; such as a continuously cultured strain in the laboratory [13] and specific to STEC O157:H7 emergence [14], evolution [15], diversity and subclassifications [16–18], short-term changes in outbreak strains [19, 20], and differences among closely related strains [21]. Sequencing has also becoming an integral part of public health responses to foodborne outbreaks [19].

The current body of knowledge concerning STEC O157:H7 genomics is growing rapidly, but up to now has lacked a comprehensive study to understand wildtype STEC O157:H7 evolution in a long-term context. Understanding how STEC O157:H7 populations change over time can provide context into strain relatedness in foodborne outbreak investigations. The US Meat Animal Research Center (USMARC) cattle research feedlot provides a unique opportunity to study STEC O157:H7 evolution in its main reservoir. Unlike most commercial cattle feedlots, where cattle are brought in from multiple locations with different background pathogens, the USMARC feedlot is a closed system that only receives cattle born and raised on the same property. This beef production system provides a unique opportunity to study STEC O157:H7 evolution in a single ecological niche while still subjected to environmental factors such as wildlife and climate. Here, we have leveraged the collection of STEC O157:H7 isolates over a 23-year period from this closed production system to study how a specific STEC O157:H7 population has evolved over time.

## Results

### Diverse subpopulations of STEC O157:H7 coexist in the same ecological niche

**Fig. 1:**
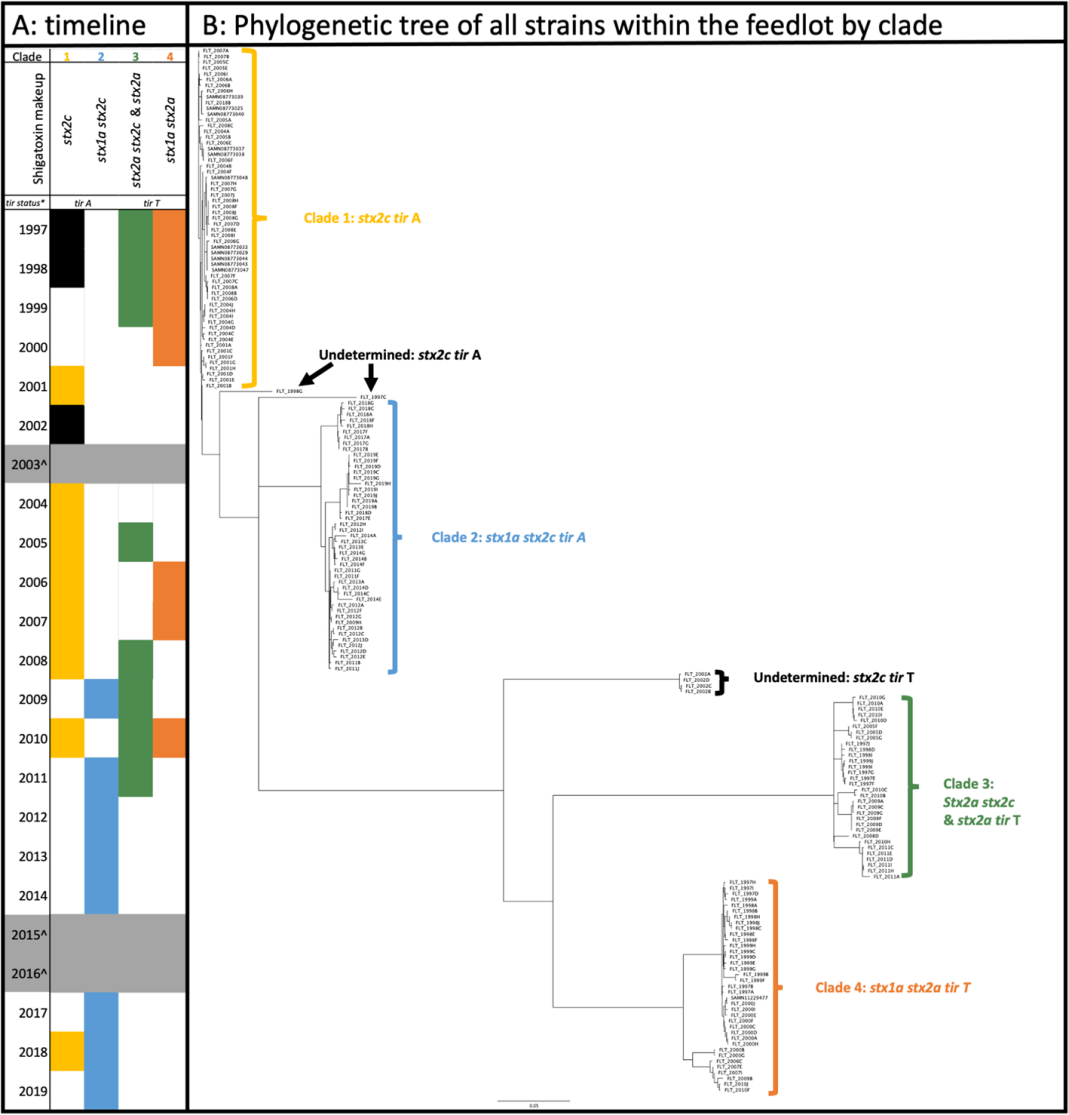
Overview of the 181 feedlot samples included in the study. (**A**) is a timeline of when clades of STEC O157:H7 were present during the retrospective study while (**B**) is a phylogenetic tree generated by Parsnp and visualized in Figtree that shows the clustering by clade and those not in a defined clade

One-hundred eighty-one (*N* = 181) STEC O157:H7 strains from 20 of the 23 years between 1997 and 2019 were included in the study. One hundred sixty-nine STEC O157:H7 strains were subjected to Illumina sequencing for the first time (with 24 within this group also sequenced with PacBio sequencing). These genomes were combined with 12 previously sequenced strains (PacBio) from the feedlot for a total of 36 total closed genomes within 181 total genomes (see Additional file 1). A phylogenetic tree was constructed from the chromosome of all strains included in the study. Four distinct clades of STEC O157:H7 were observed with six strains not classified into one of the four main clades (Fig. 1B). Clade differentiation was seen between different Shiga toxin subtypes, *tir* 255 allele type, and small plasmid (non-pO157) presence. To verify clade distribution was not driven by small plasmid presence/absence, the chromosomes of the subset of long-read closed genomes were visualized on a phylogenic tree (see Additional file 2) where the four clades were found to be intact. Within all samples, when a tree was constructed, total cluster identity among all sequences was 88.3% while clade specific trees ranged in similarity from 90.0 to 90.8%. Furthermore, when the core and pangenome of 36 closed genomes (with representation within each clade) was assessed, all strains shared 4760 genes with a pangenome made-up of 6482 genes.

Clade differentiation resulted in each clade having one to two Shiga toxin subtypes profiles: Clade 1 *stx2c*; Clade 2, *stx1a* stx2c; Clade 3 a mixed of *stx2a stx2c* or just *stx2a*; and Clade 4 *stx1a stx2a*. Clade separation was also due to *tir* 255 allele type, with Clade 1 and 2 associated with *tir* A and Clade 3 and 4 harboring *tir* T. Within the context of the four identified clades, earlier time points (1997 to 2008) saw a mix of three different clades (namely those that harbored *stx2c* [Clade 1], *stx1a stx2a* [Clade 4], and a mixed clade of *stx2a stx2c* or just *stx2a* [Clade 3]) that co-occurred throughout this period (Fig. 1A). However, 2009 saw the introduction of a clade associated with *stx1a stx2c* (Clade 2) which by 2012 through 2019 had become the dominate clade found within the feedlot. Outside of these four groups, six strains were found to not cluster within a clade, four from 2002 (*stx2c tir* T) and one from each 1997 and 1999 (both *stx2c tir* A).

In addition to the ubiquitous pO157 plasmid that occurred with all strains, four different plasmids were found within the population ranging in size from 2.7 kb to ~ 40 kb (Table 1). As previously mentioned, these plasmids occurred primarily by clade with Clade 1 harboring a 6 kb plasmid, Clade 2 harboring both a 2.7 and ~ 40 kb plasmid and Clade 4 containing a 3.3 kb plasmid (Clade 3 did not have any additional plasmids besides pO157). In terms of plasmid composition, the smaller two plasmids had periplasmic binding protein associated with nickel uptake (*nikA*) while the 6.1 kb plasmid had genes associated with mobilization (*mbeD* and *mobC*) and a type III toxin-antitoxin system. The larger plasmid (38-40 kb) contained a type II toxin-antitoxin system, type IV secretion system, and proteins associated with mobilization and regulation. Although 178 of 181 genomes did follow the pattern of clade specific plasmids, three strains did not (two from different clades lost a plasmid and one acquired a plasmid not expected in that clade) demonstrating that there was some variability with plasmid content but at a low level.

**Table 1:** Overview of non-pO157 plasmids found within the sample population. Plasmids occurred primarily within clade as noted

Plasmid	Size (bp)	Primary Clade	Previously identified in feedlot	Previously identified outside of MARC	Features of interest
1	2679	2	Yes	CP011435 (*Salmonella enterica*, 99.5% similar)	-*nikA*
2	3306	4	Yes	AB011548 (Sakai 100% similar)	-*nikA* *IsoB* and *IsoA*
3	6078	1	Yes	CP038310 (Show KS 100% similar)	-*mbeD* -*mobC* -type III toxin-antitoxin *toxN/abiQ*
4	38-40 kb	2	Yes	CP038337 (LSU61 99.1% similar)	-*cbiX* -*traD, traL, trbJ*) *-eexN* *-sprT* *-tnpB* -type II toxin-antitoxin *hicA* -type IV secretion protein *-virB11*

### Large scale genome rearrangement was observed with each clade

**Fig. 2:**
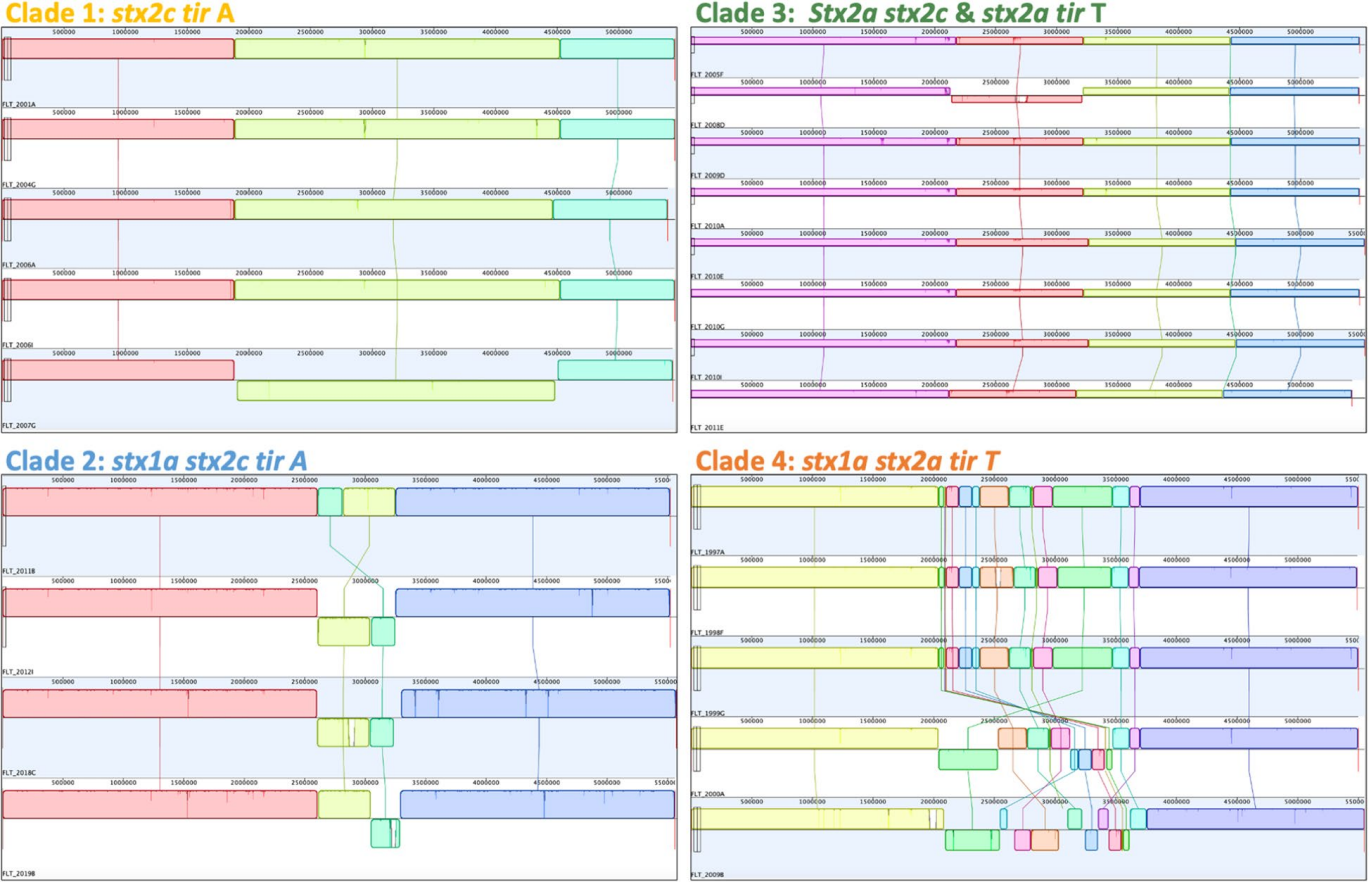
Visualization of Mauve alignments of the subset of recently sequenced closed chromosomes by clade membership. Changes in color within a panel indicate rearrangement of the chromosomes while lines within one color indicate differences of one chromosome from the others in the clade. Colored pieces of the chromosomes descending below the top line (such as the yellow piece in clade 1 FLT_2007G) indicted an inversion of that section of the chromosome in relation to others in the clade

The 24 recently sequenced long-read closed genomes were evaluated for recombination and structural differences within clade resulting in evaluation of 4 to 8 closed chromosomes per clade. All Clades exhibited some level of large-scale chromosomal rearrangement (Fig. 2). Within Clade 1 (chromosome length 5,396,494 to 5,452,894) there was one large inversion within a series of type I toxin-antitoxin system genes starting at ~ 189 kb and resulting in an approximately 262 kb inversion in the chromosome flanked by phage associated genes. Clade 2 (size 5,521,421 to 5,575,196) had a similar recombination profile, though with two pieces of chromosome displaced starting around 2605 kb into the chromosome and totaling 700 kb (the two sub pieces were around 430 kb and 270 kb in length); again, these rearrangements were all flanked by phage associated proteins. Clade 3 (size 5,423,263 to 5,527,582) had one large inversion (100 kb) flanked by phage proteins and transposase and with the start of this inversion just 1 kb upstream of *stx*2c in some cases. Clade 4 (size 5,491,538 to 5,551,813) had the highest evidence of rearrangement, with two conserved regions flanking a highly variable region of ~ 650 kb comprised of 10 subcomponents with substantial rearrangement. Across all clades and genomes, variation in chromosome size was driven by presence/absence of prophages, phage associated genes, and transposases.

### Core phageome exists along with clade specific occurrences

**Fig. 3:**
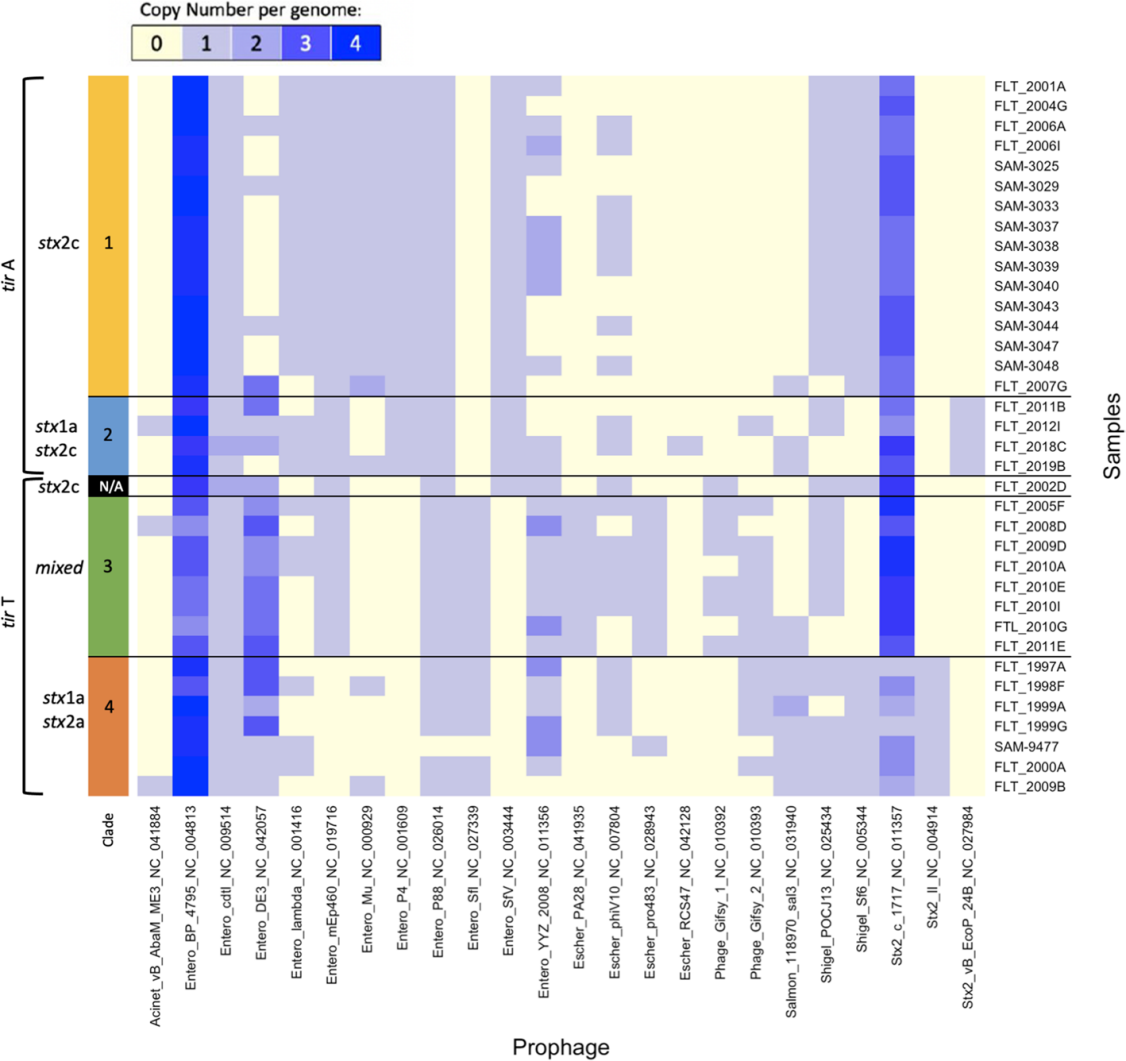
Prophages identified within all long-read samples grouped by clade and *tir* 255 SNP allele

Analysis of the 36 closed genomes identified 718 prophages; each strain contained an average of 20 phages (range 16 to 22). Three phages were found in all genomes (Fig. 3): Entero_BP_4795_NC_004813, Entero_cdlt_NC_009514, and stx2_c_1717NC_011357, with the first found in multiple copies in all genomes (note that a strain can carry the stx2_c_1717NC_011357 phage without harboring the *stx*2*c* Shiga toxin). Others were found in one *tir* 255 group, such as Entro_Sfl_NC_027339 within all *tir* T genomes (with the one exception being SAM11229477) and Entro_P4_NC_001609 in only the *tir* A allele genomes. Finally, some phages were specific or excluded from just one clade, such as Entro_mEp460_NC_019716 not found in clade 4 (*stx1a stx2a*) or Escher_PA28_NC_041935 only present in clade 3 (mixed *stx*).

### Informative SNPs are not limited to the core genome but also include mobile elements

Two thousand three hundred eighty-six polymorphisms were identified within the population via Parsnp [22]. When linkage disequilibrium (LD) was considered, the number of informative sites that captured total strain diversity was 186 on the chromosome and 23 on the pO157 plasmid, leaving 209 SNPs that tagged the total diversity across all strains. When each clade was separately analyzed, the number of informative SNPs ranged from 53 to 121, all of which had SNPs on the chromosome and pO157 with some polymorphisms also occurring on clade specific plasmids (Table 2).

**Table 2:** Number of total single nucleotide polymorphisms (SNPs) identified by Parsnp as well as number of informative SNPs after pruning variants with a LD *r*^*2*^ = 1 on the chromosome, pO157 plasmid, and in some cases clade specific plasmids

Clade	Percentage of Similarity in clade	Number of Samples in clade	Number of Variants Identified	After pruning number in Chromosome	After Pruning number in pO157	Other (plasmids specific to clade)	Total of Informative SNPs
Overall	88.3%	181	2386	186	23	N/A	209
1: *stx2c*	90.5%	59	347	118	2	1 (6.1 kb)	121
2: *stx*1a *stx*2c	90.8%	47	351	74	6	4 (40 kb)	84
3: mixed	90.0%	32	343	48	5	N/A	53
4: *stx*1a *stx*2a	90.3%	37	671	74	5	N/A	79

The 2386 polymorphisms that were trimmed down to the 209 representative SNPs were further evaluated for informativity and location. Of the unreduced set, 500 were found to be singletons (with an average of 2.7 per strain; 0.5 of those per strain [71 total] included in the 209 curated SNP set). While 77% (385/500) of the singletons were associated with non-mobile elements of the genome, the rest were on mobile elements (13% [64/500] associated with prophages and 10% [51/500] on the pO157 plasmid). These percentages were similar in the curated SNP set with 83% attributed to non-mobile elements, 10% prophage and 7% to pO157. Within the data, 18 different groupings of SNPs met the parameters of having alleles in LD *r*^*2*^ = 1.0 with those of at least three other polymorphisms (and a minimum of 5% MAF). Of those 18 groups, 14 included polymorphisms on prophages and 9 included polymorphisms on pO157 (see Additional file 3).

**Fig. 4:**
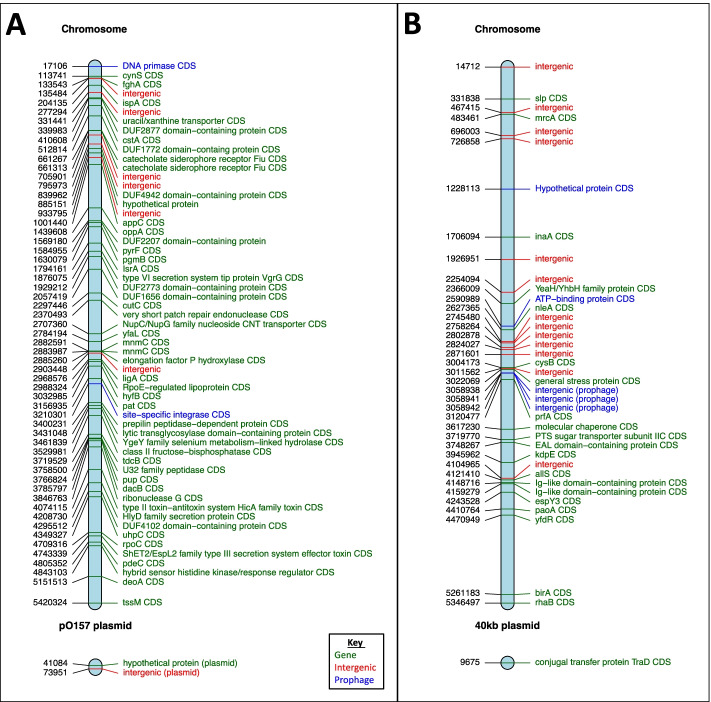
Two examples of groups of SNPs with alleles in linkage disequilibrium r^2^ = 1. Both examples contain SNPs across the chromosome (core and prophage regions) and plasmids. (**A**) is a collection of SNPs in LD across all 181 samples both within the chromosome and pO157 plasmid. (**B**) is a collection of SNPs specific to clade 2 (*stx*1a *stx*2c) and demonstrates a collection of alleles in LD both within the chromosome and within a plasmid common to the clade. Note that both examples have SNPs within both the core and prophages regions of the chromosome

Across SNP groups that were in LD *r*^*2*^ = 1.0, the majority were found to have alleles that were in LD on mobile and non-mobile elements. Two examples of this were a group of 60 polymorphisms with alleles in LD *r*^*2*^ = 1.0 within all 181 strains (Fig. 4A) and a different group of 40 polymorphism alleles specific to clade 2 (*stx1a stx2c*) in LD (*r*^*2*^ = 1.0) (Fig. 4B). Both examples contain polymorphisms in LD within mobile (prophage and plasmid) and non-mobile elements. The overall community example (Fig. 4A) has polymorphisms on both the chromosome and the pO157 plasmid while the clade specific example (Fig. 4B) illustrates variants on the chromosome and a clade specific plasmid. Additionally, both examples also illustrate the fact that polymorphism alleles in LD were found to be distributed throughout the genome and not clustered in specific regions.

### Time reconstruction of clades revealed different rates of evolution

**Fig. 5:**
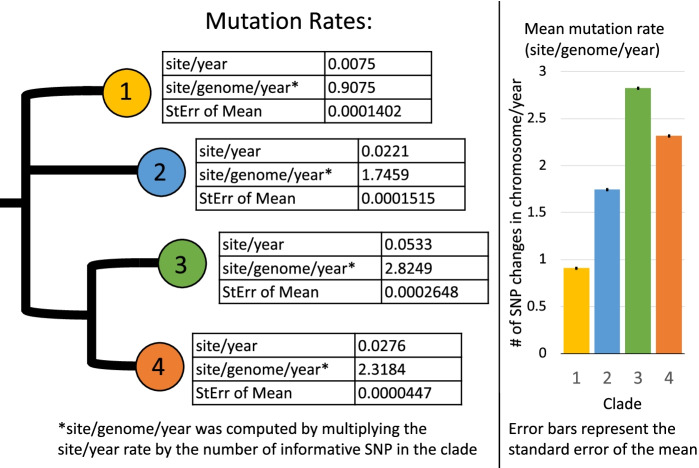
Rate of evolution as determined by time reconstruction of each major clade within the feedlot population

When a neighbor-joined tree composed of all strain informative SNPs was considered, the evolutionary rate throughout the tree was found to not be the same (*P* < 0.001). When evaluated within clade, all four clade specific trees had temporal signal as indicated by a positive correlation between genetic divergence and sampling time; however, Clade 1’s was lower (*r*^*2*^ = 0.16) when compared to the other clades (*r*^*2*^ range 0.67 to 0.87). Timed reconstruction within each clade found that each had a different rate of change (Fig. 5). All had rates of evolution between 0.9 to 2.8 SNP/genome/year. Clade 1 was by far the slowest evolving with 0.9075 site/genome/year while Clade 3 was the fastest with 2.8249 site/genome/year. When put in the context of the duration of this study, Clade 1 had only 20.87 site/genome changes over all 23 years while Clade 3 had 64.97 (Clade 2 and Clade 4 fell in between with 40.16 and 53.32 over 23 years, respectively). Rates of evolution were also compared with a constant ascertainment correction; these values ranging between 1.02 to 2.12 SNP/genome/year. While Clade 1 increased to 1.29 SNP/genome/year, Clade 2 and 4 decreased to 1.02 SNP/genome/year and 2.12 SNP/genome/year, respectively). Clade 3 differed the most from the uncorrected number with 1.61 SNP/genome/year. Regardless of ascertainment correction, Clades 1 and 2 (that had the slower evolutionary rate) both had the *tir* A allele while the two with the higher evolutionary rate, Clades 3 and 4, had the *tir* T allele.

An effort was made to understand why the rates of evolution differed by clade, which considered both recombination and replication mechanics; while growth rates of clades were of interest, there was not enough information to accurately estimate this factor. Visual inspection of a subset of complete chromosomes did not indicate that recombination had any effect on the rate of evolution. When the DNA polymerase III holoenzyme gene was specifically evaluated (due to its involvement in DNA replication) Clade 3 did have one synonymous SNP mutation (*holA* 267 G > A G) in the delta subunit of the DNA polymerase III that was not present in any of the other clades.

### Foodborne outbreak investigation tools are reproducible even with different pipelines

**Fig. 6:**
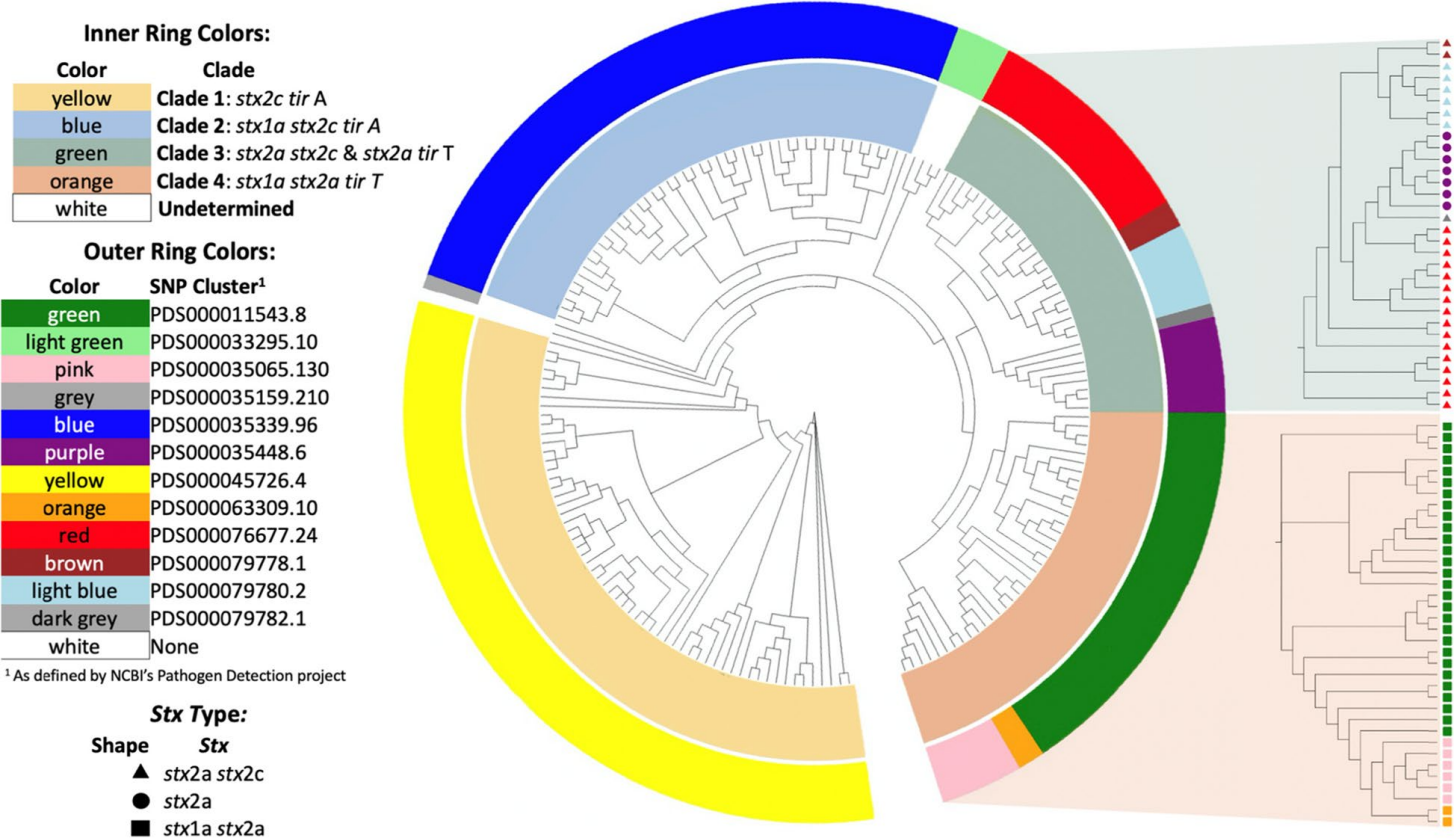
Comparison of the current study’s method (inside ring) to NCBI’s pathogen detection project (outer ring) reveled homology of results in STEC O157:H7 classification. To the right of the main tree are enlarged clades specific trees of clades 3 and 4 for greater clarity as they each contain more than one SNP cluster

Our results were compared to NCBI’s pathogen detection project’s SNP clusters and although different bioinformatic pipelines were used, classification of STEC O157 strains was consistent (Fig. 6). Clades 1 and 2 both fell into one SNP cluster each while Clades 3 and 4 divided into five and three SNP clusters, respectively. Among undetermined clades, the group from 2002 all classified into one SNP group and of the other two strains, one was assigned a SNP cluster and one was not. Within Clade 3, SNP clustering was associated with *stx* subtypes that were present. Within Clade 4, which were all *stx1a stx2a*, SNP cluster membership was visually consistent with phylogenic tree clustering. Each SNP cluster was evaluated by how many SNP differences occurred within clusters as well as the overall SNP cluster makeup (see Additional file 4). Clades 1 and 2 had a maximum SNP distance of 23 and 103, respectively. The maximum SNP differences between smaller clades that made up Clade 3 and 4 ranged from 0 to 40 with a larger group membership usually indicative of a larger max SNP distance. When comparing other strains within the NCBI defined clusters that included strains from our study, all of Clade 1’s SNP cluster was attributed to only environmental samples, the other Clade’s SNP cluster(s) varied from 59% of the cluster from clinical samples (Clade 4) to 78% (Clade 3). When short and long read libraries of the same sample were compared to each other, within the context of all other strains in the study (see Additional file 5), there was an average of 1.47 SNP differences between the long and short read libraries (range 0 to 4). Interestingly, all these differences occurred in prophage region of the genome with no SNP falling within the core genome.

## Discussion

Over 23 years, four main clades of STEC O157:H7 were present within the USMARC feedlot, each recovered over a time span of at least a decade. Persistent STEC O157:H7 colonization in cattle environments has previously been observed, such as in a study following cohorts of dairy heifers that determined through PFGE, that the same strains persisted for up to 2 years [23]. In a feedlot setting, with more transient cattle populations, the evidence for resident versus transient strains is not as definitive. A characterization of STEC O157 from one feedlot noted that indistinguishable restriction endonuclease digestion patterns (REDPs) were isolated over several years in the same feedlot, pointing towards a resident strain and suggesting incoming cattle with diverse pathogens were less important to the overall STEC population [24]. On the other hand, a study of multiple feedlot cattle also using PFGE found that although some strains were feedlot specific, their low number in conjunction with high number of diverse patterns within the same dataset pointed toward the likelihood strains were specific to incoming cattle groups and not resident to the feedlot [25]. Nonetheless, a recent genomic characterization of STEC O157:H7 in multiple cow-calf herds, where some farms were sampled multiple times, concluded that strains of STEC O157:H7 may be persistent for months on the same farm [26]. The data presented here, with each clade present for at least a decade, makes a strong argument that resident strains can be harbored in the same feedlot; with the obvious limitation that outside cattle were not introduced into this system thus limiting competition between resident and transient strains. Of equal importance to note is that six strains were found in the feedlot that did not fall into a major clade, perhaps highlighting the result of when a less competitive strain is introduced from an outside source and is unable to outcompete the resident strains. This introduction could come from wildlife, birds, insects or other means as there are two feedlots within 2 miles of the Center’s boundary and 5 and 10 miles from the feedlot.

Just as interesting as the persistence of strains, is the genomic diversity. The *tir* T allele has been previously associated with human clinical samples (vs. bovine) when compared to the *tir* A allele, suggesting that these *tir* T strains have a higher propensity to cause disease (and that the *tir* A allele maybe indicative of a bovine host niche) [10]. Another facet of diversity is Shiga toxin type (*stx1* and/or s*tx2*), *stx2* is known to be more virulent than *stx1* alone or in combination with each other, and more specifically that *stx2a* is more virulent than *stx2c* [27]. In the context of these two factors, while not the majority, there is a subset of *tir* T *stx2a* strains within Clade 3 that are considered more likely to cause human disease and be more virulent than other strains in this population. This highlights the fact that although the feedlot is closed to outside cattle, there wasn’t a single strain that was predominant over the entire period but there were diverse strains, including some more likely to cause human disease.

An unexpected finding that cannot be fully examined here is the shift from three dominant Clades (1, 3 and 4) in the late 1990’s through early 2000’s to the emergence and subsequent dominance of Clade 2. The retrospective nature of this study prohibits further exploration into the reasons behind the change in STEC O157:H7 clades, although it should be noted that sampling schemes were not standardized across year or pen location. It is noteworthy however, that the introduction of wet distillers grains with solubles (WDGS) into the rations happens to correspond with the emergence and dominance of Clade 2 strains. The addition of WDGS into the feed ration beginning in 2012 corresponds with the shift from three strains to one predominant strain. While WDGS has been documented to increase O157 in feedlot cattle [28–30] there is no data specific to corroborate if WDGS promotes a specific strain of O157 over others. Further work is now planned in the feedlot to provide a more systemic understanding of the different variables that can affect STEC O157:H7 strain persistence or loss.

Large chromosomal rearrangements were observed within each Clade with rearrangements varying from one inversion (Clade 1) to complex rearrangement (Clade 4). Genomic plasticity in STEC O157:H7 has been well-documented [31, 32] and was not unexpected. In this study, rearranged chromosomal pieces were flanked by known transposase and phage associated genes, this was also true when a single chromosome had an insertion or deletion not shared by other chromosomes in the clade. Neither the number of informative SNPs that defined a Clade or the rate of change over time appeared to be correlated with rearrangements based on the limited subset of closed genomes that were evaluated from each clade.

*E. coli* and STEC have been associated with high prophage content in the chromosome, with two estimates of 13.5 and 14.6% of the chromosome attributed to prophages in STEC O157:H7 and 8.7 to 16.7% in STEC O26:H11 [33, 34]. Other work describing a much more diverse set of 56 STEC O157:H7 genomes (J Bono and M Weinroth, personal observation) found the mean number of prophages in STEC O157:H7 to be the same as well as describing Enterobacteria phage BP-4795 as ubiquitous to all STEC O157:H7 strains in the study and Stx2-converting phage 1717 and Shigella phage POCJ13 to be present in more than 95% of the STEC O157:H7 population. A study of multiple serotypes of STEC found Enterobacteria phage BP-4795 and Stx2-converting phage 1717 to also be the most common within the chromosome [35]. The high occurrence of these two phages in the population could be due to both containing a putative integrase gene that is associated with the integration of phage DNA into bacterial chromosomes through lysogenic infection [36]. Here, three prophages were shared by all strains which was expected as the population of STEC O157:H7 was highly homologous and as a result more similarity between strains were expected. Clade specific prophages were also observed with the most apparent being the specific presence of *stx* prophage by clade. This was not surprising, given that specific prophages have been associated with specific *stx* genes in the past [37, 38]. Other prophages that were specific to clades or linked with *tir* alleles likely contributed to some of the phylogenetic differences observed between clades.

Here, multiple SNP alleles were found to be in complete LD across the core genome, phages, and plasmids. This was of interest as we expected SNP alleles in LD to mainly reside on the core genome, as mobile elements are traditionally associated with transient roles and not ubiquitous in populations. The appearance of informative SNPs on the pO157 was expected, given recent work linking this plasmid to the chromosome from an evolutionary perspective [39]. More surprising was the observation that some alleles were found to be in complete LD across core and prophage regions. There could be several reasons for this including: coevolution due to functionality pressure, a selection sweep resulting in hitchhiking genes, a bottle neck, or that LD relationships were established prior to the clonal expansion described over these 23 years. Unfortunately, most tools specific to selection sweep or LD decay are more appropriate on much larger datasets and attempts to modify them to test these hypotheses were not successful. While further examination (either bioinformatically or in the wet lab) is needed to understand the cause of these relationships, we still find the complete LD between core genome SNP alleles and phages and plasmids of interest.

BEAST [40], the method used here to determine rate of evolutionary changes, has been previously used to determine rate of change in both pathogenic *E. coli* and other bacteria of interest. Past estimates of enterotoxigenic *E. coli* [41] and specifically STEC O157:H7 [42] evolution have found similar rates of evolution (2 to 2.5 and 95% highest posterior density [HPD] of 2.4 to 2.8 SNPs/genome/year, respectively). *Shigella sonnei* was found to have a similar rate of mutation 2.2 SNPs/genome/year in the core genome (95% HPD 1.8 to 2.6) [43]. Both *E. coli* and *Shigella sonnei* studies also found different mutation rates between linages [41, 43]. It is unclear why different strains of the same serotype would have different rates of evolution in the same stable environmental niche. In terms of why mutation rates differ, recombination, differences in DNA replication, and generation interval were considered. Visual inspection of a subset of closed genomes did not reveal any recombination events that associated with rate of evolution. When considering high fidelity DNA replication, DNA polymerase III holoenzyme was specifically evaluated as it is the enzyme primarily responsible for replicative DNA synthesis. One synonymous mutation was found on the DNA polymerase III subunit delta (*holA*) only in Clade 3 (*holA* 267 G > A G). Although synonymous mutations can cause changes to protein expression and function [44], the fact that the other three Clades investigated were identical means this change was not likely a contributor to evolution rate differences. While not collected in this study, further experimentation on growth rate could be explored in the future as a possible driver in SNP differences (i.e. that the clade with the most SNPs was the fastest growing) but ultimately there was not enough information (such as optical density measurements or environmental factors such as pen floor diversity and moisture) to accurately predict generation intervals.

Because STEC O157:H7 is a pathogen of public health interest in foodborne outbreaks, we conducted two additional comparisons to contextualize our findings to other publicly available data. The first comparison was to classify all strains from our study with NCBI’s Pathogen Detection (PD) SNP clusters. The NCBI Pathogen Detection is a centralized database for sequenced pathogens from food, human infections, and the environment, with samples contributed from many U.S. public health agencies as well as some international agencies [45]. Due to the breadth of this database (as of this writing there were 170,622 strains within the *E. coli*/ *Shigella* group) it allowed for a more global perspective of the relatedness of the strains in this study. Clades 1 and 2, the two slowest evolving, were each found to belong to just one SNP Clade each. While, both Clade 3 and 4 belonged to several SNP clusters, though these differences were driven by *stx* group and some core structural differences which were reflected on the phylogenetic tree.

Interestingly, although Clades 1 and 2 were found to both belong to only one SNP cluster each, this study found the maximum SNP difference in these Clades to be 121 and 84, respectively, while the PD found the maximum SNP difference of those same clades to be 23 and 103, these differences are likely due to different bioinformatic pipelines of the two methods. This paired with the fact that the PD reported the average SNP differences in Clade 1 as 11 but Clade 2 as 45, indicates that while this study determined Clade 1 to be more diverse, the PD found the opposite was true. While from a research perspective this is of interest, the public health implications are not as clear. While SNP cluster PDS000045726.4, which Clade 1 is a part of, is clearly a niche to the USMARC feedlot (the only strains included are from that location), SNP cluster PDS000035339.96, of which Clade 2 strains are members, harbors almost 200 additional strains, with 146 of those from clinical settings. Pragmatically, both analyses grouped these strains in the same way with the main drivers being *stx* genes presence. The other two Clades were composed of multiple SNP clusters and were easily discernable by *stx* inclusion and distance on a phylogenic tree.

The other comparison that was made was the difference between genomes assembled from only short reads (Illumina) or a combination of short and long-reads (PacBio with Illumina reads to polish the completed whole genome) from the same DNA extraction. While an average of 1.47 SNP discrepancy was found between each pair, all these SNP were in prophage regions. There were not any informative SNPs found between the short and long read pairs in the core genome. Because tracebacks during an outbreak often are only focused on the core genome, this indicates that both long and short read genomes of good quality can be used interchangeable in an outbreak investigation. However, in cases of highly related strains in an outbreak, inclusion of the mobile elements can increase differentiation power. For example, isolates from two outbreaks had different phage types (PT) but only differed from one another by three core SNP [20]. The addition of long read sequencing to close the outbreak genomes identified addition SNPs, insertion of bacteriophages unique to each strain and one strain gaining a plasmid that not only contained multiple drug resistance genes but also a previously unknown mechanism for acquiring resistance to group 3 typing phages that resulted in the different PT. These results and those in the current study demonstrate that complete closed genomes not only gives the genetic relatedness of strains but also characterizes micro-evolution events that allows us to learn more about the dynamic nature of STEC O157:H7 genome evolution.

## Conclusions

This study leveraged a unique closed-system cattle feedlot to study the evolution of STEC O157:H7 populations over 23 years. Genomic analysis revealed that four main clades of STEC O157:H7 resided in the feedlot with greater than 96% of the population falling into one of these clades. Differentiation of clades could be attributed to *stx* composition, *tir* 255 alleles, and small plasmid presence. Large scale chromosomal rearrangements were present in all clades and mainly flanked by transposase and genes associated with mobile elements. Further examination of representative SNPs found that alleles in LD were not confined to the core genome alone but included both plasmids and prophages. Rates of evolution between clades differed from less than 1 SNP within the chromosome annually to 2.8, though no definitive reason was found for these differences. These findings offer several insights into the evolution of this foodborne pathogen. Firstly, that rate of change, even within serotype and ecological niche, can vary between strains; a noteworthy finding with implications for long-term pathogen tracking as it relates to public health. Furthermore, the relationship between alleles in LD across core and mobile elements, while still under investigation, leads to questions on when elements traditionally considered mobile (such as prophage or plasmids) should be considered integrated within the genome to the extent that they are classified as a part of the core genome. Finally, comparisons with the NCBI’s pathogen detection pipeline, showed that the phylogenetic trees generated in this study were almost identical for Clades 1 and 2, which demonstrated the lowest evolutionary rates in individual SNP clusters, while Clades 3 and 4 with the highest evolutionary rates, were found to be members of multiple SNP clusters.

## Methods

### Description of sample population

At its peak during the year, USMARC has approximately 14,000 cattle with half that amount housed in a feedlot. Unlike most commercial feedlots, USMARC is a closed system, meaning there is no introduction of cattle born off site. This has been maintained since the creation of the Center in the late 1970’s and early 1980’s. As a result, this facility offers the opportunity to study a beef production system with very little influx of STEC O157:H7 except through environmental factors such as wildlife. STEC O157:H7 strains isolated from feedlot feces (from a mix of fecal grabs and pen surface material) collected from 1997 to 2019, that were previously confirmed as STEC O157:H7, were identified for inclusion in this study. In a retrospective survey, up to ten strains from each sample year (see Additional file 6) were included for a total of 181 strains (see Additional file 7), some years did not have all ten strains and 3 years (2003, 2015, and 2016) did not have any. Strains were chosen by taking one from each independent sampling taken from the feedlot during a calendar year. The most independent sampling take during a year was ten, so this became the maximum number of strains used per year.

### Short-read library construction and sequencing

Frozen characterized strains were struck onto CHROMagar O157 (CHROMagar Microbiology) and grown overnight in 3.5 mL of tryptic soy broth (Becton Dickinson) at 37 °C for DNA isolation. One milliliter of growth media was used in the QIAamp DNA mini kit extraction kit (QIAGEN) according to manufacturer instructions with the addition of 4ul of RNase A (100 mg/ml) after lysis to each sample. DNA extractions were quantified and checked for purity using a Nanodrop 2000 spectrophotometers (Thermo Scientific) with 20 mL/ng as the minimum acceptable concentration. DNA (1.5 μg) from each sample was sheared to 350 bp with a Covaris Sonicator S220 (Covaris) using Covaris microtube (Covaris) and used as an input in the TruSeq DNA PCR-Free High Throughput Library Prep Kit (Illumina). DNA libraries were quantified via qPCR with the KAPA Library Quant Kit for Illumina libraries (Roche) and diluted to 4 nM prior to pooling (two pools of 96). Each pool was run on an Illumina NextSeq with the Mid-Output Kit (2 × 150).

### Short read bioinformatics

Raw reads were concatenated across lanes so there was one forward and one reverse file per sample. Concatenated samples were run in the Shovill pipeline (https://github.com/tseemann/shovill) using the trim option with a minimum contig size of 200 bp. Briefly, raw reads were trimmed of adaptors, assembled with SPAdes (version 3.12.0) [46], and error corrected with Pilon (version 1.22) [47]. Contigs assembled by Shovill were imported into Geneious Prime (2020.1.2; Biomatters Ltd., Auckland, New Zealand) and typed for the *tir* allele [10] via the classify tool and *stx* subtype and O and H antigen using the VirulenceFinder 2.0 and SerotypeFinder 2.0, respectively (http://www.genomicepidemiology.org/). Contigs were constructed into a phylogenetic tree with Parsnp version 1.2 [22] with default settings and CP038428.1 as the reference. The tree was visualized with FigTree version 1.4.3 [48].

### Long-read library construction and sequencing

Twenty-four phylogenetically diverse strains were grown individually in 10 mL of Luria broth (Becton Dickinson) overnight at 37 °C in a shaking incubator. After overnight growth, 250 mL of inoculum was transferred to a fresh 10 mL of LB where it was incubated for 3 hours under the same incubation conditions. DNA from the culture was extracted via the QIAGEN Genomic tip 500/G kit via manufacture instructions. Ten micrograms of total DNA were sheared using the Covaris G-tubes spun at 4200RPM then 4100RPM. Libraries were constructed using the PacBio SMRTbell Express Template Preparation Kit 2.0 (Pacific Biosciences). Non-competing barcoded samples were pooled in groups of 4 to 5 and sized selected on a Blue Pippin (Sage Science) High Pass DNA Gel Cassette using a 15 kb protocol. Final library pools were quantified and run on the PacBio Sequel II. In addition to the 24 strains sequenced here, 12 strains from a previous study underwent the same procedure.

### Long-read bioinformatics

Raw reads were assembled in SMRT Link’s v. 8.0 Microbial Assembly pipeline (using HGAP3 [49]). Generated contigs were imported into Geneious Prime 20.1.2 (Biomatters, Ltd., Auckland, New Zealand) and chromosomes and plasmids were circularized and reoriented to a common origin. Illumina libraries from the same strains were used for polishing the genomes with Pilon [47]. Finally, Illumina reads where mapped to the chromosome and known plasmids using Geneious (Biomatters, Ltd.) and unused reads were de novo assembled (also in Geneious) for small plasmid identification. All genomes and plasmids were annotated with the NCBI Prokaryotic Genome Annotation Pipeline [50]. Prophages were identified in all long-read samples with PHASTER [51]. Plasmid presence was visualized with the heatmap3 package (version 1.1.9) [52] in R (version 4.0.2). The pan and core genomes were assessed with ROARY (version 3.11.2) [53] after annotation with Prokka (version 1.14.5) [54].

### Identification of plasmids in short-read libraries

Outside of the pO157 plasmid, four plasmids were identified in the long-read samples. A fasta file for each clade was generated that contained the closed chromosome and corresponding pO157 plasmid of the oldest isolate from that clade and the four additional plasmids identified by long-read sequencing. These fasta files were used as a reference to identify small plasmids not identified by long-read sequencing. Trimmed reads (from the Shovill output) from each short-read library were aligned to the reference with bwa version 0.7.17 [55] and plasmid presence was determined by the pileup script from bbmap version 1.9 [56] with 99% coverage used as the presence threshold. Non-assigned contigs were interrogated for identification of further plasmids by reviewing contigs from these reads and comparing them to published plasmids was well as considering the contig quality (coverage depth, read length, and number of reads used).

### Evaluation of large-scale genome rearrangement

Previously generated GenBank annotation files of the 24 most recently sequenced closed chromosomes were included in this comparison. Each clade’s strains (which varied in number from 4 to 8) were visualized with the progressive Mauve aligner (1.1.3) [57] in Geneious (Biomatters, Ltd.). Alignments were visually inspected with specific interest in inversions (indicated by a locally collinear block (LCB) of the chromosome reversed in relation to the same LCB in other strains) and lack of uniformity (indicated by a LCB being located in a different place on the chromosome with relation to other strains).

### Identification of SNPs and pruning

A Parsnp (version 1.2) [22] tree of all genomes (chromosome and all plasmids) was constructed using SAMN11229477 as the root, because it was the oldest isolate from the feedlot. The resulting .grr file that contained all the variant sites was converted to a .vcf using Harvest tools [22]. The .vcf file was imported into PLINK (version 1.90) [58] and the linkage disequilibrium based variant pruner option “--indep-pairwise” with a window size the entire length of all SNPs identified with an r^2^ = 0.99 (and visually inspected to assure r^2^ = 1) was used to reduce all SNPs to just those that were informative. This process was repeated for each of the four clades identified for a total of five sets of informative SNPs that capture the minimum set of informative SNPs (not in LD) able to define the diversity of each clade and the strain set as a whole. Linkage disequilibrium SNPs associations were visualized with the LinkageMapView (version 2.1.2) [59] in R.

### Time reconstruction and rate of changes estimates

A tree composed of all informative SNPs (as described above) concatenated into a multiple alignment file from all samples was constructed using the Neighbor-Joining method in MEGA X (version 10.2) [60] where evolutionary distances were computed using the Maximum Composite Likelihood method and rate variation among sites was modeled with a gamma distribution. A molecular clock test was performed by comparing the maximum likelihood value for the given topology with and without the molecular clock constraints under Tamura-Nei model (+G) and differences in evolutionary rates among sites were modeled using a discrete Gamma (G) distribution.

All four clades were assessed for temporal signal with TempEst (version 1.5.3) [61] and found to have positive r^2^ values thus suitable for analysis timed reconstruction. Clade specific fasta multiple alignment files with only informative SNPs were used as input to BEAST v1.10.4 [40] with the following parameters: gamma (G4) site model with HKY and a strict clock and a coalescent constant population, three independent runs of 100 M with a 10% burn in were conducted and log files were combined. Clade 1 did not have an appropriate effective sample size (ESS), so the chain was increased to 500 M which provided an acceptable ESS. All models were also run with a constant ascertainment correction (https://groups.google.com/g/beast-users/c/V5vRghILMfw) through editing the .xml file (Additional file 8). The invariant sites were determined by converting the Parsnp generated .ggr file to an alignment file with the command harvesttools -i parsnp.ggr -M parsnpLCB.aln from Harvest suite v1.2. The .aln file was use with the python script countInvSites.py (https://github.com/GonzaloYebra/misc_scripts/blob/master/countInvSites.py) to determine the invariant sites for editing the .xml files.

Recombination rates and the sequence of the DNA polymerase III holoenzyme were compared between the clades as an attempt to understand different rates of change between clades. For evaluation of recombination rates, a subset of two complete chromosomes from each clade (for a total of 8 chromosomes) were aligned via Mauve [57]. Visual inspection of the alignment did not reveal any clade specific recombination. DNA polymerase III SNP differences were also evaluated using the same subset of eight annotated chromosomes, genes associated with DNA polymerase III were excised from each genome and concatenated for one alignment per genomes. A multiple alignment of the 8 genomes was constructed with the Geneious Prime base multiple aligner. The alignment was visuality inspected for polymorphisms. Upon finding one polymorphism, a 50 bp segment of each variation was extracted and used via classify on all closed genomes to confirm that the polymorphism was not limited to the subset of genomes originally interrogated.

### Contextualizing results in a food safety perspective

Because of the public health implications associated with *E. coli* O157:H7, two additional analyses were performed to provide additional context to the strains. First, all closed genomes and contigs assembled from those not closed were submitted to the NCBI Pathogen Detection (ncbi.nlm.nih.gov/pathogens) for SNP cluster assignment. These SNP clusters were compared to this study’s clade differentiation visually with Evolview v3 [62]. For comparison of PacBio and Illumina sequencing method similarity, a phylogenetic tree using all samples in the study plus the short read contigs in addition to the long read closed genomes was constructed with Parsnp v1.2 [22] and the resulting grr file was converted to a vcf using Harvest tools [22] for individual SNP comparison.

## Supplementary Information

**Additional file 1.** Phylogenic tree 181 strains sequenced. Parsnp was used to generate the phylogenetic relationship and the tree was visualized in FigTree. The strain’s names in red indicates the strains that have complete closed genomes.

**Additional file 2.** Phylogenic tree visualized in FigTree and constructed via Parsnp of only chromosomes of the 36 strains subjected to long-read (PacBio) sequencing. Coloring indicates previously delineated clade structure and confirms clade membership was not due to plasmid presence.

**Additional file 3 **Variants found to be in *r*^2^ = 1 linkage disequilibrium with at least three other variants (and a minimum of 5% MAF) across all samples (inclusive of both the chromosome and associated plasmids). Variant locations are described in context to reference genome CP038428.

**Additional file 4.** Summary of the results from each SNP cluster detected in the study via the Pathogen Detection database. Organization is first by clade identify within this study then the pathogen detection database SNP cluster membership. Sub-branch information is only associated with samples from this study and overall SNP cluster is inclusive of all strains in the SNP cluster inclusive of study strains.

**Additional file 5.** Phylogenic tree visualized in FigTree and constructed via Parsnp of all samples in the study with both the long read (FLT_*) and short read (FLT_SR_*) samples from the same DNA pool. Coloring is by sample pair with the number to the right indicating the number of informative SNP differences found between each pair (with the average being 1.47 between pairs).

**Additional file 6.** Number of strains collected by year. Note that a 2007 study met the inclusion criteria for this study and as a result a larger number of strains are included in that year.

**Additional file 7.** Strains used in study with genotype data, sorted first by long- and short-read then by clade.

**Additional file 8.** Text file containing the .xml information for the four clades that were generated in Beauti, manually edited for ascertainment bias correction and imported into Beast1 to generate evolutionary rate estimates.

## Data Availability

The datasets supporting the conclusions of this article are available in the National Center for Biotechnology Information (NCBI) repository under BioProject accession numbers PRJNA627032, https://www.ncbi.nlm.nih.gov/search/all/?term=PRJNA627032; PRJNA528413, https://www.ncbi.nlm.nih.gov/search/all/?term=PRJNA528413; and PRJNA445267, https://www.ncbi.nlm.nih.gov/search/all/?term=PRJNA445267. Additional file 7 contains the individual strain’s NCBI GenBank and SRA accession numbers.

## Abbreviations

ESS: Effective sample size
HUS: Hemolytic-uremic syndrome
LB: Luria broth
LCB: Locally collinear block
LD: Linkage disequilibrium
MAF: Minor allele frequency
NCBI: National Center for Biotechnology Information
PacBio: Pacific Biosciences
PD: NCBI Pathogen Detection
PFGE: Pulsed-field gel electrophoresis
PT: Phage type
REDP: Restriction endonuclease digestion pattern
SNP: Single nucleotide polymorphism
STEC: Shiga toxin-producing *Escherichia coli*
*stx*: Shiga toxin
*tir* T: *tir* 255 T > A T allele
*tir* A: *tir* 255 T > A A allele
USMARC: US Meat Animal Research Center
WDGS: Wet distillers grains with solubles
