# Supplementary material for: Rates of evolutionary change of resident Escherichia coli O157:H7 differ within the same ecological niche
Source: BMC Genomics. 2022 Apr 7;23:275. doi: 10.1186/s12864-022-08497-6 (PMC8991562; doi:10.1186/s12864-022-08497-6)
Supplement: Supplementary file 1 — Additional file 1. Phylogenic tree 181 strains sequenced. Parsnp was used to generate the phylogenetic relationship and the tree was visualized in FigTree. The strain’s names in red indicates the strains that have complete closed genomes. [file 12864_2022_8497_MOESM1_ESM.docx]

**
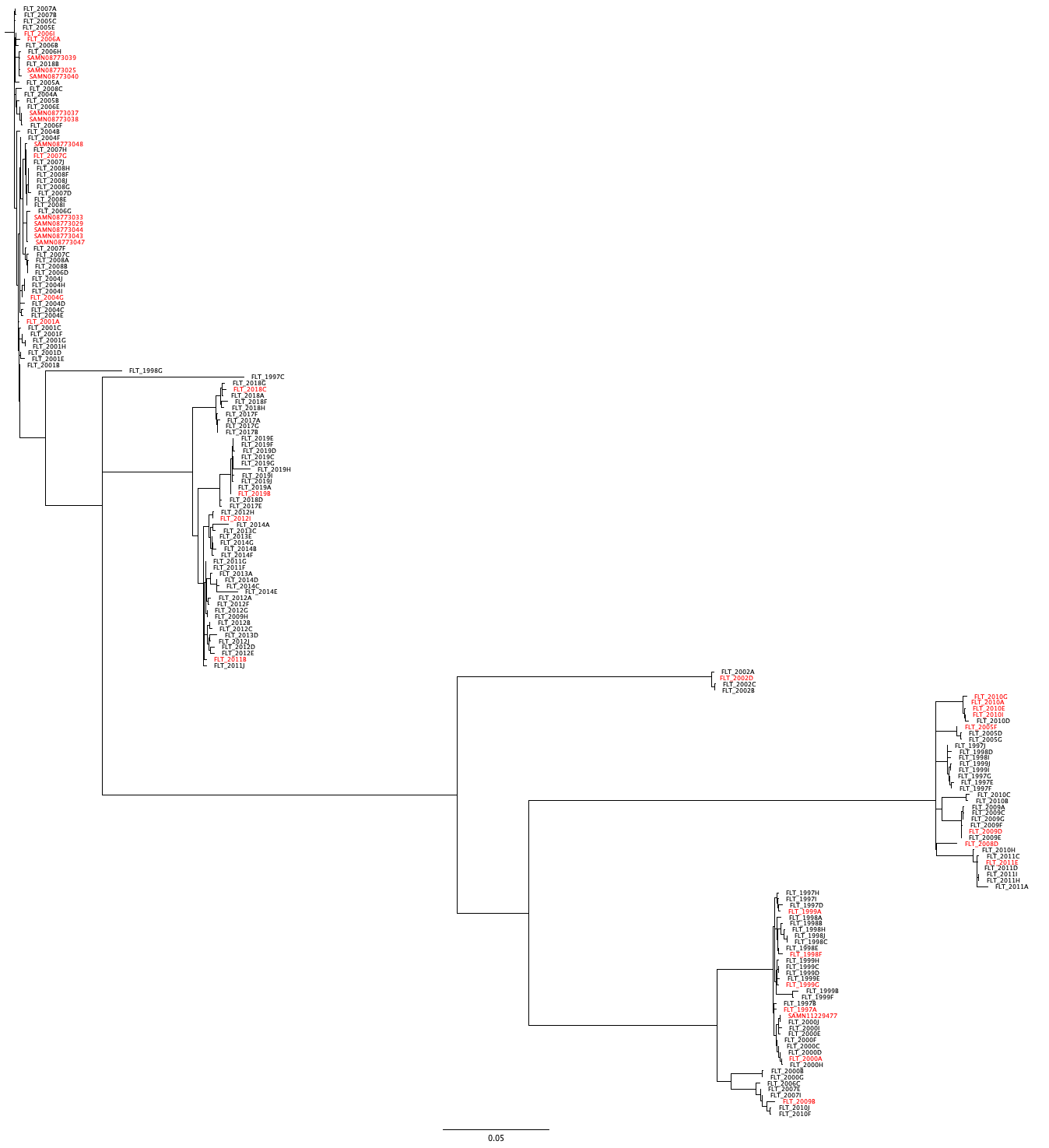
**

**Additional File 1.** Phylogenic tree visualized in FigTree and constructed via Parsnp of all 181 strains sequenced. The strain’s names in red indicates the strains that have complete closed genomes.
