# Supplementary material for: Rates of evolutionary change of resident Escherichia coli O157:H7 differ within the same ecological niche
Source: BMC Genomics. 2022 Apr 7;23:275. doi: 10.1186/s12864-022-08497-6 (PMC8991562; doi:10.1186/s12864-022-08497-6)
Supplement: Supplementary file 2 — Additional file 2. Phylogenic tree visualized in FigTree and constructed via Parsnp of only chromosomes of the 36 strains subjected to long-read (PacBio) sequencing. Coloring indicates previously delineated clade structure and confirms clade membership was not due to plasmid presence. [file 12864_2022_8497_MOESM2_ESM.docx]

**Additional File 2.** Phylogenic tree visualized in FigTree and constructed via Parsnp of only chromosomes of the 36 strains subjected to long-read (PacBio) sequencing. Coloring indicates previously delineated clade structure and confirms clade membership was not due to plasmid presence.
