# Supplementary material for: Rates of evolutionary change of resident Escherichia coli O157:H7 differ within the same ecological niche
Source: BMC Genomics. 2022 Apr 7;23:275. doi: 10.1186/s12864-022-08497-6 (PMC8991562; doi:10.1186/s12864-022-08497-6)
Supplement: Supplementary file 3 — Additional file 3 Variants found to be in r2 = 1 linkage disequilibrium with at least three other variants (and a minimum of 5% MAF) across all samples (inclusive of both the chromosome and associated plasmids). Variant locations are described in context to reference genome CP038428. [file 12864_2022_8497_MOESM3_ESM.docx]

**Additional File 3.** Variants found to be in *r*^2^=1 linkage disequilibrium with at least three other variants (and a minimum of 5% MAF) across all samples (inclusive of both the chromosome and associated plasmids). Variant locations are described in context to reference genome CP038428.

| Location | ID of first variant on genome | Total Number of variants in (LD *r*^2^=1) | Number of variants on core genome | Number of variants on pO157 | Number of variants in prophage  regions |
| --- | --- | --- | --- | --- | --- |
| 3,152 | CAAAGTCCGA.AAGGCCGGGA | 90 | 4 | 0 | 86 |
| 6,770 | TTCGACTCCT.ATTATCGGCA | 11 | 10 | 0 | 1 |
| 9,883 | ATGATTCGAG.CATTGAAAAT | 325 | 303 | 5 | 17 |
| 17,106 | GTGCCGCAGG.GTTTAATCCT | 60 | 54 | 2 | 2 |
| 22,702 | GCAGAGCGGG.GGAGACGTTG | 95 | 86 | 1 | 8 |
| 25,780 | AACAGAAAAT.GCTACCCCAC | 165 | 151 | 13 | 1 |
| 29,723 | AGGGCAAAGG.TGTAGGAGGC | 70 | 67 | 2 | 1 |
| 30,905 | CCTCAAGCTG.CTCGGTTTTG | 26 | 23 | 0 | 3 |
| 39,222 | TTTTCATTGT.ATTTCTTCCC | 278 | 254 | 6 | 18 |
| 44,340 | AAATTTGAAT.AGATGTATTA | 28 | 25 | 1 | 2 |
| 164,450 | CTACAAGGGA.TCTGCGTCAC | 60 | 54 | 1 | 3 |
| 287,503 | GCACCACCTC.CGGCGTGGAA | 24 | 23 | 0 | 1 |
| 338,005 | CAATAAGGGG.TTCTCATGTT | 7 | 7 | 0 | 0 |
| 563,024 | TAAAACAAAT.TACACCGATA | 3 | 2 | 0 | 1 |
| 1,869,980 | TTGAATATTC.TGAGAAATAT | 3 | 3 | 0 | 0 |
| 1,989,651 | GGTAATTAAG.GAGTTCTCCA | 3 | 0 | 0 | 3 |
| 2,368,046 | CTCCCCACCT.TCTTTTTTAT | 3 | 3 | 0 | 0 |
| 3,462,290 | AACGGCGTAT.CTATCATGGG | 6 | 6 | 0 | 0 |
