# Supplementary material for: Rates of evolutionary change of resident Escherichia coli O157:H7 differ within the same ecological niche
Source: BMC Genomics. 2022 Apr 7;23:275. doi: 10.1186/s12864-022-08497-6 (PMC8991562; doi:10.1186/s12864-022-08497-6)
Supplement: Supplementary file 4 — Additional file 4. Summary of the results from each SNP cluster detected in the study via the Pathogen Detection database. Organization is first by clade identify within this study then the pathogen detection database SNP cluster membership. Sub-branch information is only associated with samples from this study and overall SNP cluster is inclusive of all strains in the SNP cluster inclusive of study strains. [file 12864_2022_8497_MOESM4_ESM.docx]

**Additional File 4.** Summary of the results from each SNP cluster detected in the study via the Pathogen Detection database. Organization is first by Clade identify within this study then the pathogen detection database SNP cluster membership. Sub-branch information is only associated with samples from this study and overall SNP cluster is inclusive of all strains in the SNP cluster inclusive of study strains.

|  |  |  | Sub-branches with study samples | | |  | Overall SNP Cluster | | |  | Isolation Source | |
| --- | --- | --- | --- | --- | --- | --- | --- | --- | --- | --- | --- | --- |
| Clade | SNP Cluster Name | Succeeded by^1^ | Number | Max SNP distance | Average SNP distance |  | Number | Max SNP distance | Average SNP distance |  | Clinical | Environmental |
| Clade 1 | PDS000045726.4 | -- | 47 | 23 | 11 |  | 69 | 25 | 13 |  | 0 | 69 |
| Clade 2 | PDS000035339.96 | -- | 45 | 103 | 45 |  | 234 | 117 | 58 |  | 146 | 88 |
| Clade 3 | PDS000076677.24 | PDS000076677.32 | 11 | 40 | 17 |  | 1584 | 40 | 17 |  | 1244 | 340 |
|  | PDS000079778.1 | -- | 2 | 3 | 3 |  | 12 | 62 | 30 |  | 10 | 2 |
|  | PDS000079780.2 | -- | 6 | 2 | 1 |  | 6 | 2 | 1 |  | 0 | 6 |
|  | PDS000079782.1 | -- | 1 | -- | -- |  | 2 | 3 | 3 |  | 0 | 2 |
|  | PDS000035448.6 | -- | 7 | 9 | 4 |  | 13 | 46 | 20 |  | 5 | 8 |
| Clade 4 | PDS000011543.8 | -- | 26 | 16 | 8 |  | 30 | 18 | 8 |  | 0 | 30 |
|  | PDS000063309.10 | PDS000083645.1 | 2 | 0 | 0 |  | 216 | 105 | 42 |  | 50 | 166 |
|  | PDS000035065.130 | PDS000083644.2 | 6 | 20 | 11 |  | 345 | 118 | 59 |  | 299 | 46 |
| Undetermined | PDS000033295.10 | -- | 4 | 0 | 0 |  | 31 | 52 | 16 |  | 23 | 8 |
|  | PDS000035159.210 | PDS000035159.217 | 1 | -- | -- |  | 688 | 119 | 60 |  | 659 | 29 |
| ^1^ During our study, some SNP clades assigned by the pathogen detection database were updated with new ID’s, both are presented here for clarity. | | | | | | | | | | | | |
