# Supplementary material for: Rates of evolutionary change of resident Escherichia coli O157:H7 differ within the same ecological niche
Source: BMC Genomics. 2022 Apr 7;23:275. doi: 10.1186/s12864-022-08497-6 (PMC8991562; doi:10.1186/s12864-022-08497-6)
Supplement: Supplementary file 5 — Additional file 5. Phylogenic tree visualized in FigTree and constructed via Parsnp of all samples in the study with both the long read (FLT_*) and short read (FLT_SR_*) samples from the same DNA pool. Coloring is by sample pair with the number to the right indicating the number of informative SNP differences found between each pair (with the average being 1.47 between pairs). [file 12864_2022_8497_MOESM5_ESM.docx]

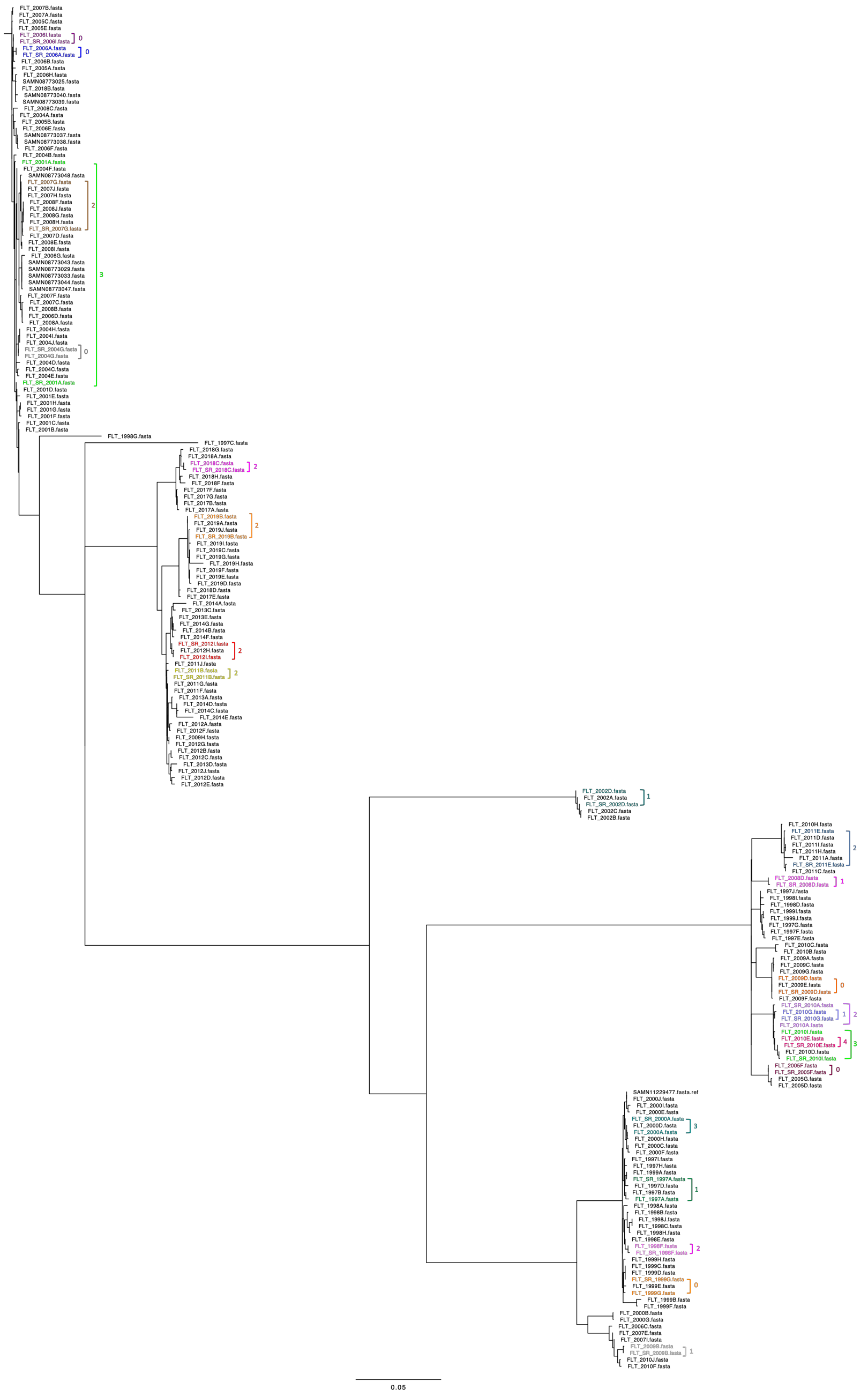


**Additional File 5.** Phylogenic tree visualized in FigTree and constructed via Parsnp of all samples in the study with both the long read (FLT_*) and short read (FLT_SR_*) samples from the same DNA pool. Coloring is by sample pair with the number to the right indicating the number of informative SNP differences found between each pair (with the average being 1.47 between pairs).
