# Supplementary material for: Rates of evolutionary change of resident Escherichia coli O157:H7 differ within the same ecological niche
Source: BMC Genomics. 2022 Apr 7;23:275. doi: 10.1186/s12864-022-08497-6 (PMC8991562; doi:10.1186/s12864-022-08497-6)
Supplement: Supplementary file 6 — Additional file 6. Number of strains collected by year. Note that a 2007 study met the inclusion criteria for this study and as a result a larger number of strains are included in that year. [file 12864_2022_8497_MOESM6_ESM.docx]

**Additional File 6.** Number of strains collected by year. Note that a 2007 study met the inclusion criteria for this study and as a result a larger number of strains are included in that year.

| **Year** | **Strain** **Count** |
| --- | --- |
| 1997 | 10 |
| 1998 | 10 |
| 1999 | 11 |
| 2000 | 10 |
| 2001 | 8 |
| 2002 | 4 |
| 2003 | 0 |
| 2004 | 10 |
| 2005 | 7 |
| 2006 | 9 |
| 2007 | 21 |
| 2008 | 10 |
| 2009 | 8 |
| 2010 | 10 |
| 2011 | 10 |
| 2012 | 10 |
| 2013 | 4 |
| 2014 | 7 |
| 2015 | 0 |
| 2016 | 0 |
| 2017 | 5 |
| 2018 | 7 |
| 2019 | 10 |
| **Total** | **181** |
